# Control of mechanical pain hypersensitivity in mice through ligand-targeted photoablation of TrkB-positive sensory neurons

**DOI:** 10.1038/s41467-018-04049-3

**Published:** 2018-04-24

**Authors:** Rahul Dhandapani, Cynthia Mary Arokiaraj, Francisco J. Taberner, Paola Pacifico, Sruthi Raja, Linda Nocchi, Carla Portulano, Federica Franciosa, Mariano Maffei, Ahmad Fawzi Hussain, Fernanda de Castro Reis, Luc Reymond, Emerald Perlas, Simone Garcovich, Stefan Barth, Kai Johnsson, Stefan G. Lechner, Paul A. Heppenstall

**Affiliations:** 1 Epigenetics and Neurobiology Unit, EMBL Rome, Via Ramarini 32, Monterotondo, 00015 Italy; 2 Molecular Medicine Partnership Unit (MMPU), Heidelberg, Germany; 3 Institute of Pharmacology, Heidelberg University, Im Neuenheimer Feld 366, Heidelberg, 69120 Germany; 4 Department of Gynecology and Obstetrics, University Hospital RWTH Aachen, Pauwelsstrasse 30, Aachen, 52074 Germany; 5 Ecole Polytechnique Federale de Lausanne, Institute of Chemical Sciences and Engineering (ISIC), Institute of Bioengineering, National Centre of Competence in Research (NCCR) in Chemical Biology, Lausanne, 1015 Switzerland; 6 Institute of Dermatology, Catholic University of the Sacred Heart, Largo A. Gemelli 8, Rome, 00168 Italy; 7 South African Research Chair in Cancer Biotechnology, Institute of Infectious Disease and Molecular Medicine (IDM), Department of Integrative Biomedical Sciences, Faculty of Health Sciences, University of Cape Town, Anzio Road, Observatory, 7925 South Africa; 8 Department of Chemical Biology, Max-Planck Institute for Medical Research, Heidelberg, 69120 Germany

## Abstract

Mechanical allodynia is a major symptom of neuropathic pain whereby innocuous touch evokes severe pain. Here we identify a population of peripheral sensory neurons expressing TrkB that are both necessary and sufficient for producing pain from light touch after nerve injury in mice. Mice in which TrkB-Cre-expressing neurons are ablated are less sensitive to the lightest touch under basal conditions, and fail to develop mechanical allodynia in a model of neuropathic pain. Moreover, selective optogenetic activation of these neurons after nerve injury evokes marked nociceptive behavior. Using a phototherapeutic approach based upon BDNF, the ligand for TrkB, we perform molecule-guided laser ablation of these neurons and achieve long-term retraction of TrkB-positive neurons from the skin and pronounced reversal of mechanical allodynia across multiple types of neuropathic pain. Thus we identify the peripheral neurons which transmit pain from light touch and uncover a novel pharmacological strategy for its treatment.

## Abstract (web summary)

There are several classes of sensory neuron that contribute to pain states. Here, the authors demonstrate that TrkB^+^ sensory neurons detect light touch under normal conditions in mice but contribute to hypersensitivity in models of chronic pain, and that ligand-guided laser ablation of TrkB^+^ sensory neurons in the mouse skin attenuates this hypersensitivity.

## Introduction

In neuropathic pain patients, hypersensitivity to light touch can develop to the extent that movement of a single hair shaft is sufficient to provoke severe pain^1^. This is difficult to treat using conventional analgesics such as opioid or NSAIDS, and it impacts greatly upon quality of life due to the pervasive nature of mechanical stimuli; for example, small movements of the body, or the weight of clothing can cause severe pain in neuropathic patients^2^. While much recent progress has been made in delineating the spinal circuits that gate mechanical pain^3–6^, the identity of the sensory neurons that input this sensation into the spinal cord is less clear^7^.

Hypothetically, mechanical hypersensitivity could be mediated either by sensitization of nociceptors (hyperalgesia), or through integration of input from low-threshold mechanoreceptors into pain transmitting circuits (allodynia)^1^. In human studies, there is little evidence for nociceptor sensitization, and most reports indicate that mechanical allodynia is conveyed by myelinated A-fiber mechanoreceptors, although it is not known which subtype^2^. Thus, differential block of these nerves alleviates brush-evoked pain^8^, and the short latency of pain perception is indicative of the fast conduction velocity of A-fibers^9^. In experimental animal studies the situation is less clear. For example, it has been demonstrated that mice develop mechanical allodynia in neuropathic pain models even when all nociceptors are genetically ablated^10^. In contrast, isolectin B4-positive nociceptors have also been implicated as drivers of allodynia^11^. Another study proposed unmyelinated C-low threshold mechanoreceptors marked by Vglut3 expression as a candidate population for driving mechanical hypersensitivity^12^. However, allodynia persists even when these neurons fail to develop^13^, and recent evidence indicates that transient Vglut3 expression in spinal interneurons accounts for the phenotype^4^. Finally, A-fibers marked by de novo expression of neuropeptide Y^14^, TLR5 expression^15^, or TrkB expression^16^ have also been suggested to influence mechanical hypersensitivity.

The skin is innervated by an array of functionally distinct populations of mechanoreceptors that can be distinguished by their conduction velocity, response properties, mechanical threshold, and the type of end organ that they innervate^17^. Molecular markers for many of these populations have recently been identified, among which, the receptor tyrosine kinase TrkB, has been proposed to mark both D-hairs^18^ and rapidly adapting Aβ mechanoreceptors (RAMs)^19–21^. These neurons innervate the same hair follicle forming a functional unit^18^, and are among the most sensitive of all cutaneous mechanoreceptors, and respond preferentially to dynamic stimuli, making them a strong candidate for the neuronal subtype mediating mechanical allodynia.

Here we generated an inducible TrkB^CreERT2^ mouse line to manipulate this population of neurons in adult mice in vivo. Through loss- and gain-of-function experiments, we demonstrate that TrkB-positive neurons are both necessary and sufficient for the expression of mechanical hypersensitivity after nerve injury. Because TrkB is a cell surface receptor, we reasoned that its ligand BDNF could allow for pharmacological targeting of these neurons and suppression of their role in driving mechanical allodynia. We therefore generated a recombinant BDNF^SNAP^ fusion protein and conjugated it to the near infra-red (IR) photosensitizer IRDye®700DX phthalocyanine (IR700). In vivo delivery of BDNF^SNAP^-IR700 and near IR illumination led to a retraction of TrkB-positive sensory neurons from the skin and a prolonged alleviation of mechanical hypersensitivity in multiple models of neuropathic pain. Thus, ligand-guided laser ablation of a subset of mechanoreceptors in the skin is an effective means of selectively silencing the input, which drives mechanical allodynia in neuropathic pain states.

## Results

### TrkB-positive sensory neurons are D-hair and RAMS

**Fig. 1:**
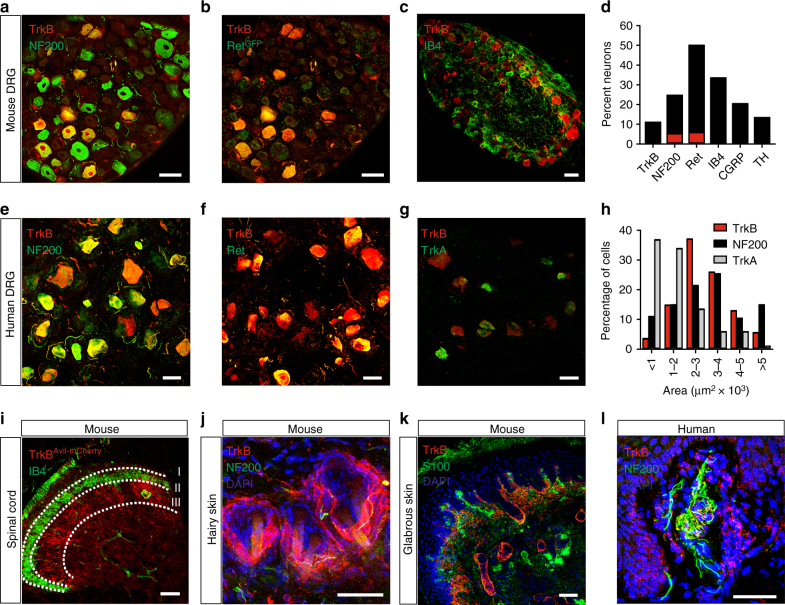
TrkB-positive sensory neurons are putative mechanoreceptors. **a**–**d** Double immunofluorescence of DRG sections from TrkB^CreERT2^::Rosa26^RFP^ mice with **a** NF200, **b** Ret, visualized using TrkB^CreERT2^::Rosa26^RFP^::Ret^EGFP^ triple transgenic mice, **c** IB4, **d** quantification of staining on mouse DRG sections; TrkB^+^ cells account for ~10% of all DRG neurons and all co-express NF200 or NF200 + Ret^eGFP^, while they are negative for IB4, CGRP, and TH. **e**–**h** Double immunofluorescence of human DRG sections stained with antibodies against TrkB and **e** NF200, **f** Ret, **g** TrkA. **h** Size distribution for human DRG neurons expressing TrkB, NF200, and TrkA. **i** Section through the lumbar spinal cord of TrkB^CreERT2^::Avil^mCherry^ mice stained with IB4. **j** TrkB^+^ lanceolate endings in a section of the back hairy skin of TrkB^CreERT2^::Rosa26^SnapCaaX^ labeled with Snap Cell TMRstar (red), NF200 (green), and DAPI (blue). **k** Section from the glabrous skin of TrkB^CreERT2^::Rosa26^ChR2YFP^ (red) stained with anti-S100 a marker for Meissner’s corpuscles (green) and DAPI (blue) showing TrkB^+^ innervation. **l** Section from human glabrous skin stained with antibodies against TrkB (red) and NF200 (green), and DAPI (blue). Scale bars, **a**–**c** and **i** 50 μm, **e**–**g** and **j**–**l** 40 μm.

We generated TrkB^CreERT2^ mice (Supplementary Fig. 1) and crossed them with Rosa26^RFP^ reporter mice to examine co-localization of TrkB with established cellular markers in adult mouse sensory ganglia. Approximately 10% of neurons in the dorsal root ganglia (DRG) were positive for TrkB^CreERT2^, corresponding to the ~8% of cells which expressed TrkB mRNA (Fig. 1d and Supplementary Fig. 2). Expression was evident in large neurons marked by NF200, and NF200 plus Ret, and not present in nociceptors positive for IB4 or CGRP, or C low threshold mechanoreceptors marked by TH (Fig. 1a–d and Supplementary Fig. 3). To assay TrkB^CreERT2^-positive sensory input into the spinal cord, we generated a reporter line in which Cre-dependent expression of mCherry was driven from the sensory neuron-specific Avil locus (Supplementary Fig. 1B, C). TrkB^CreERT2^::Avil^mCherry^-positive sensory neurons were present in laminae III/IV of the dorsal horn of the spinal cord, where they formed column-like structures extending dorsally (Fig. 1i)^18^. We further investigated the projections of TrkB neurons to the skin. TrkB^CreERT2^ fibers formed longitudinal lanceolate endings around hair follicles (Fig. 1j) and extended to Meissner corpuscles in the glabrous skin (Fig. 1k). We also examined expression of TrkB in human tissue using a TrkB antibody. In agreement with mouse data, TrkB immunoreactivity was present in human DRG in large neurons co-expressing NF200 and Ret, but largely absent from nociceptors expressing TrkA (Fig. 1e–h). Similarly, in glabrous skin, TrkB immunoreactivity was detected in NF200-positive fibers innervating Meissner corpuscles (Fig. 1l). Collectively, these data indicate that TrkB^CreERT2^ marks a population of putative mechanoreceptive neurons in mouse and human.

**Fig. 2:**
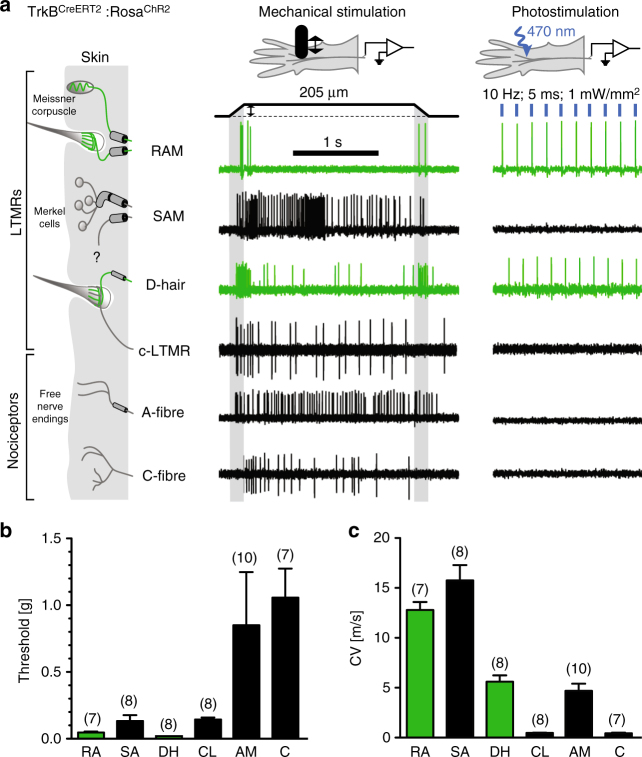
TrkB-positive sensory neurons are myelinated low-threshold mechanoreceptors. In vitro skin nerve preparation from TrkB^CreERT2^::Rosa26^ChR2^ mice showing **a** representative responses to 10 Hz stimulation with blue light, **b** the minimal force required to elicit an action potential in the indicated fiber type, and **c** the conduction velocities of the fiber types. Green bar represents TrkB^+^ afferents, *n* number indicated in brackets. Error bars represent SEM

To unequivocally establish the identity of TrkB^CreERT2^-positive sensory neurons, we characterized their response properties utilizing a combination of electrophysiology and optogenetic activation. Mice expressing the light-gated ion channel channelrhodopsin-2 (ChR2) in TrkB-positive cells were generated (TrkB^CreERT2^::Rosa26^ChR2^) and an ex vivo skin nerve preparation was used to identify neuronal subtypes which could be concomitantly activated by light. Strikingly, we determined that all D-hair and RAMs could be stimulated by light, whereas all other subtypes of sensory neurons were not responsive (Fig. 2a–c). Thus, TrkB marks myelinated neurons that innervate hair follicles and Meissner’s corpuscles and are tuned to detect gentle mechanical stimuli.

### TrkB^+^ neurons detect light touch under basal conditions

**Fig. 3:**
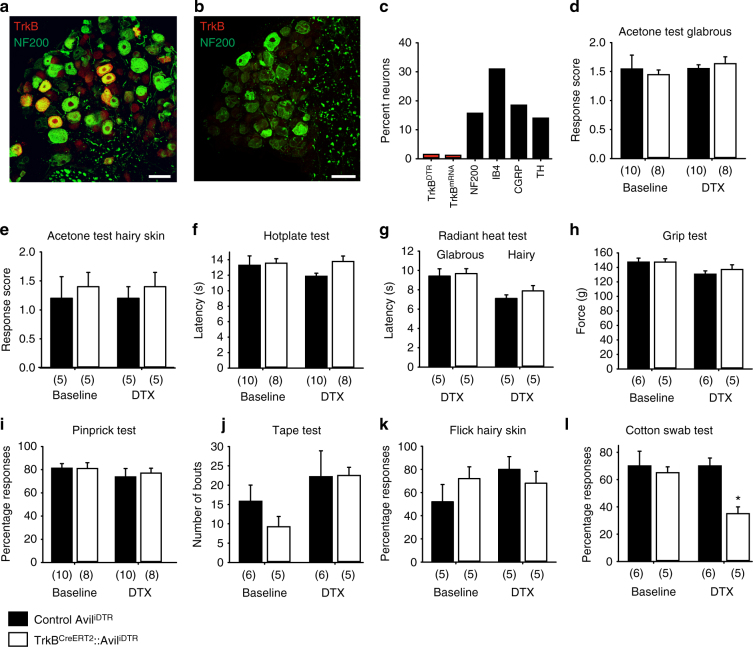
Diphtheria toxin-mediated ablation of TrkB^+^ sensory neurons. Immunostaining of DRG sections of TrkB^CreERT2^::Avil^iDTR^ mice with an antibody against the diphtheria toxin receptor (red) from **a** untreated mice and **b** after i.p. injections of diphtheria toxin. **c** Quantification of DRG sections indicating a ~90% decrease in TrkB^DTR^ and TrkB^mRNA^ cells after ablation and 36% reduction in NF200^+^ neurons without affecting other subpopulations. **d**–**l** Behavioral responses in littermate control mice (Avil^iDTR^, black bars) and TrkB^CreERT2^::Avil^iDTR^ mice (white bars) showing no differences in responses before and after ablation in the acetone drop test (*t*-test; *p* = 0.800) on **d** glabrous and **e** hairy skin, **f** hot plate test (*t*-test; *p* = 0.914), **g** radiant heat test on glabrous and hairy skin (*t*-test; *p* = 0.263), **h** grip test (*t*-test; *p* = 0.484), **i** pin-prick test (*t*-test; *p* = 0.691), **j** tape test (*t*-test; *p* = 0.776), and **k** punctate mechanical flick test applied to the hairy back skin (*t*-test; *p* = 0.196). **l** Ablated mice show a reduction in sensitivities to cotton swab (*t*-test, *p* = 0.002). Scale bars in **a**, **b** 50 μm, error bars indicate SEM

**Fig. 4:**
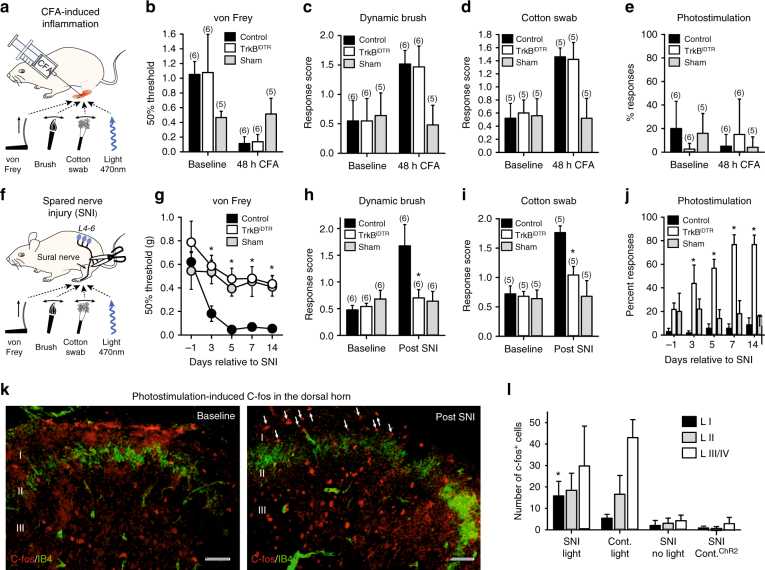
TrkB^+^ neurons are necessary and sufficient to convey mechanical allodynia after nerve injury. **a** Schematic of CFA injection and behavior tests following ablation of TrkB^+^ neurons. Mechanical hypersensitivity in control Avil^iDTR^ (black bar), TrkB^CreERT2^::Avil^iDTR^ (white bar), and sham-injected (gray bar) mice 48 h after CFA injections as measured by **b** von Frey filaments (*t*-test, *p* = 0.886), **c** dynamic brush stimuli (*t*-test; *p* = 0.537), and **d** cotton swab stimuli (*t*-test; *p* = 0.242). All mice received two diphtheria toxin injections 7 days and 10 days before CFA treatment. **e** Paw withdrawal frequencies in control (Rosa26^ChR2^ mice without Cre, black bar), and CFA-injected (white bar) or sham (grey bar)-injected paw of TrkB^CreERT2^::Rosa26^ChR2^ mice upon stimulation with 473 nm blue light. No significant differences under baseline conditions and 48 h after CFA injection (Mann–Whitney test; *p* = 0.886). **f** Schematic of SNI and behavioral tests following ablation of TrkB^+^ neurons. **g** von Frey mechanical thresholds indicating that ablation of TrkB^+^ neurons abolished the development of mechanical allodynia after SNI in TrkB^CreERT2^::Avil^iDTR^ mice (white circles) as compared to Avil^iDTR^ controls (black circles) (*n* = 7 for both sets, two-way RM ANOVA; *p* = 0.001 followed by a Bonferroni post hoc test). Sham-operated mice (grey circles) did not develop mechanical hypersensitivity. **h**, **i** Reduced dynamic allodynia in ablated TrkB^CreERT2^::Avil^iDTR^ mice (white bar) as compared to littermate controls (black bar; *t*-test *p* = 0.016) stimulated with a **h** brush or **i** cotton swab. Sham-operated mice did not develop dynamic allodynia. **j** Nociceptive behavior evoked by optogenetic stimulation of the paws of Rosa26^ChR2^ mice without Cre (black bars) and TrkB^CreERT2^::Rosa26^ChR2^ (white bars) mice after SNI, or ipsilateral paws (grey bars) of sham-operated mice (two-way RM ANOVA; *p* = 0.001). **k** Cross sections of lumbar spinal cord from TrkB^CreERT2^::Rosa26^ChR2^ mice labeled for c-fos (red) and IB4 (green) after 1 min exposure to 15 Hz blue light. Representation of section taken from an uninjured mouse and a section from an injured mouse at 7 days post SNI. **l** Quantification of the number of c-fos positive cells in laminae I, II, and III/V of the lumbar spinal cord within a 40 μm section. Data are shown for SNI, non-injured and sham-operated TrkB^CreERT2^::Rosa26^ChR2^ mice with or without light, and control SNI Rosa26^ChR2^ mice without Cre. Baseline indicates pre-ablation and pre-treatment. Error bars indicate SEM. Scale bars in **k**, 40 μm

To determine the role played by TrkB^CreERT2^-positive D-hairs and RAMs in sensory evoked behavior, we genetically ablated TrkB neurons in the peripheral nervous system. We used a Cre-dependent diphtheria toxin receptor driven from the Avil locus^22,23^ that allowed for selective deletion of TrkB-positive neurons only in adult sensory ganglia. Upon systemic injection of diphtheria toxin we achieved an ~85% ablation of TrkB^CreERT2^::Avil^iDTR^ and TrkB mRNA-positive neurons with a reduction in the number of NF200-positive neurons by 36% (presumably reflecting an additional loss of TrkB neurons not detected by histological methods), and no change in the expression of other markers (Fig. 3a–c, Supplementary Fig. 2, and Supplementary Table 1). We performed a series of behavioral tests in these animals examining sensory responses to a range of thermal and mechanical stimuli applied to the glabrous and hairy skin. There were no differences in responses to evaporative cooling evoked by acetone application (Fig. 3d, e), or in thresholds to noxious heat (Fig. 3f, g) after diphtheria toxin ablation. Similarly, grip strength (Fig. 3h) was unaltered by ablation of TrkB^CreERT2^ neurons, as were responses to noxious pinprick (Fig. 3i), and static or punctate mechanical stimulation of the hairy skin of the back (Fig. 3j, k). We further examined responses to dynamic mechanical stimuli by monitoring responses to brushing of the plantar surface of the paw. Using a puffed out cotton swab which exerts forces in the range 0.7–1.6 mN, we observed a significant reduction in responsiveness upon ablation of TrkB-positive neurons (Fig. 3l). Intriguingly, these differences were not apparent upon application of stronger dynamic forces using a paint brush (>4 mN, Fig. 4b). Thus, under basal conditions, TrkB-positive sensory neurons are required for behavioral responses to the lightest of mechanical stimuli.

### TrkB neurons signal pain from light touch after nerve injury

On account of the exquisite sensitivity of TrkB-positive neurons, we next asked whether they contribute to mechanical hypersensitivity in models of injury-induced pain. We took both a loss-of-function approach using genetic ablation, and a gain-of-function approach using optogenetic activation of TrkB neurons. We first considered a model of inflammatory pain by injecting complete Freund’s adjuvant (CFA) into the plantar surface of the paw, and monitoring responses to von Frey filaments and dynamic brush or cotton swab stimuli (Fig. 4a). Ablation of TrkB neurons in TrkB^CreERT2^::Avil^iDTR^ mice had no effect on any of these measures of mechanical hypersensitivity after inflammation (Fig. 4b–d). We next induced neuropathic pain in mice using the spared nerve injury (SNI) model (Fig. 4f). Control mice developed a profound mechanical and cold hypersensitivity in the sural nerve territory of the paw (Fig. 4g–i and Supplementary Fig. 4). Strikingly, upon ablation of TrkB^CreERT2^::Avil^iDTR^ sensory neurons, mice did not develop mechanical allodynia to either punctate or brushing stimuli (Fig. 4g–i), while response to cold stimuli was unchanged (Supplementary Fig. 4). We further examined whether optogenetic activation of TrkB neurons could evoke pain behavior. Using photo-stimulation parameters which evoked robust firing in the ex vivo skin nerve preparation, we observed no discernible behavioral response to light application to the paw either in basal conditions or after CFA-induced inflammation in TrkB^CreERT2^::Rosa26^ChR2^ mice (Fig. 4e and Supplementary Movie 1). Identical stimulation conditions applied to the hairy skin of the ear auricle evoked a brief ear twitch in TrkB^CreERT2^::Rosa26^ChR2^ mice (Supplementary Movie 2), likely reflecting activation of the dense network of mechanoreceptors in this structure^24^. We performed further experiments in mice with the SNI model of neuropathic pain. Three days after injury we observed that selective stimulation of TrkB neurons with light-evoked nocifensive behavior. This was evident as a prolonged paw withdrawal from the stimulation, lifting of the paw and licking of the illuminated area (Fig. 4j and Supplementary Movie 3) that continued for several minutes after light application. Such behavior persisted throughout the 2 weeks' observation period and was never observed in control mice (Fig. 4j). Thus, under neuropathic pain conditions, TrkB sensory neurons are necessary and sufficient to convey the light touch signal that evokes pain.

As a neuronal correlate of this apparent pain behavior, we examined induction of the immediate early gene C-fos in the dorsal horn of the spinal cord (Fig. 4k, l and Supplementary Fig. 5A–B). In TrkB^CreERT2^::Rosa26^ChR2^ mice without injury, optical stimulation evoked C-fos immunoreactivity primarily in laminae III and IV of the spinal cord, the region where TrkB neurons terminate (Fig. 4k, l). Upon nerve injury, however, identical stimulation parameters induced C-fos staining in lamina I of the dorsal horn (Fig. 4k, l), an area associated with nociceptive processing. To determine whether this resulted from aberrant sprouting of TrkB^+^ afferents into superficial laminae after nerve lesion, we examined TrkB^+^ sensory input into the spinal cord using TrkB^CreERT2^::Avil^mCherry^ mice (Supplementary Fig. 5C, D). We were unable to detect any difference in TrkB distribution in mice with SNI, suggesting that de novo expression of C-fos likely arises from plasticity within the interneuron network of the dorsal horn^3–5,25^ and not from sprouting^26,27^ of TrkB^+^ mechanoreceptors into the superficial dorsal horn.

### Ligand-guided laser ablation TrkB^+^ sensory neurons

**Fig. 5:**
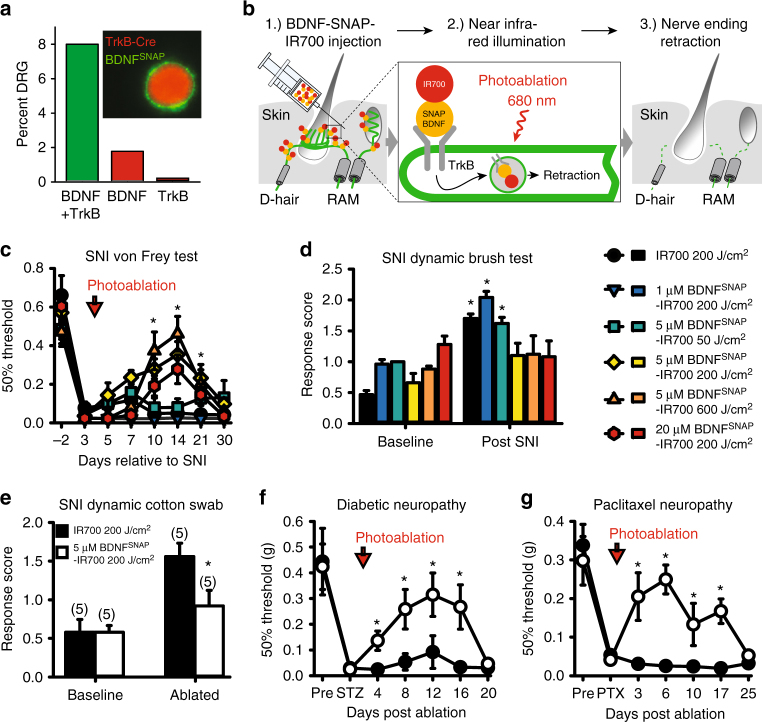
Optopharmacological targeting of TrkB^+^ neurons with BDNF^SNAP^. **a** Labeling (inset) and quantification of dissociated DRG from TrkB^CreERT2^::Rosa26^RFP^ mice with BDNF^SNAP^ shows substantial overlap of BDNF^SNAP^ binding to TrkB^+^ cells (*n* = 4, 450 cells). **b** Schematic representation of BDNF^SNAP^-IR700 injection and photoablation. **c** BDNF^SNAP^-IR700-mediated photoablation of the paw of SNI mice results in a dose-dependent reversal of mechanical hypersensitivity as assayed with von Frey filaments (*n* = 10, two-way RM ANOVA; *p* = 0.003 followed by a Bonferroni post hoc test) and **d** dynamic brush stimuli (*t*-test; *p* = 0.016). **e** Hypersensitivity to cotton swab is also reversed by photoablation (*t*-test: *p* = 0.042). **f** BDNF^SNAP^-IR700-mediated photoablation reverses mechanical allodynia in the streptozotocin (STZ) model of diabetic neuropathy (*n* = 5, two-way RM ANOVA; *p* = 0.007 followed by a Bonferroni post hoc test. Open circles; 5 µM BDNF^SNAP^-IR700 at 200 J/cm^2^, closed circles, 5 µM IR700 at 200 J/cm^2^). **g** BDNF^SNAP^-IR700-mediated photoablation reverses mechanical allodynia in the paclitaxel (PTX) model of chemotherapy-induced neuropathy (*n* = 5, two-way RM ANOVA; *p* = 0.027 followed by a Bonferroni post hoc test. Open circles; 5 µM BDNF^SNAP^-IR700 at 200 J/cm^2^, closed circles, 5 µM IR700 at 200 J/cm^2^). Error bars indicate SEM

In light of the clinical importance of mechanical allodynia in neuropathic pain patients, we sought to develop a pharmacological strategy to exploit the striking selectivity of TrkB to the peripheral neurons, which provoke this pain state. We reasoned that BDNF, the ligand for TrkB, may give access to these neurons and allow for their manipulation in wild type, non-transgenic animals. To this end we produced recombinant BDNF protein with an SNAP-tag fused to its C terminus that would enable its chemical derivatization. BDNF^SNAP^ was labeled in vitro with fluorescent SNAP-Surface 647 substrate and applied to HEK293T cells expressing neurotrophin receptors. Fluorescently labeled BDNF^SNAP^ displayed remarkable selectivity for its cognate receptor complex TrkB/p75, and did not bind to cells expressing related neurotrophin receptors TrkA/p75 or TrkC/p75 (Supplementary Fig. 6A–C). We further tested whether BDNF^SNAP^ would recognize native TrkB receptors in DRG neurons. BDNF^SNAP^ was conjugated to Qdot 655 quantum dots and applied to dissociated DRG from TrkB^CreERT2^::Rosa26^RFP^ mice. We observed a >95% overlap between BDNF^SNAP^- and TrkB^CreERT2^-positive cells (Fig. 5a), indicating that recombinant BDNF^SNAP^ is a highly selective means of targeting TrkB neurons.

To manipulate TrkB neurons in vivo, we reasoned that BDNF^SNAP^ may allow for targeted photoablation of these neurons through delivery of a photosensitizing agent^28,29^. We synthesized a benzylguanine-modified derivative of the highly potent near infrared photosensitizer IRDye®700DX phthalocyanine (IR700) and conjugated it in vitro to BDNF^SNAP^. In initial experiments we applied BDNF^SNAP^-IR700 to HEK293T cells expressing TrkB/p75 and assayed cell death following near infra-red illumination. In cells expressing TrkB/p75, we observed substantial cell death 24 h after brief illumination that was not evident upon mock transfection or treatment with IR700 alone (Supplementary Fig. 6D–F). We next sought to assess the therapeutic potential of this approach by investigating the effects of BDNF^SNAP^-IR700-mediated photoablation in wild-type mice with neuropathic pain. Upon establishment of robust mechanical allodynia 3 days after SNI, we injected a range of concentrations of BDNF^SNAP^-IR700 into the ipsilateral paw of injured mice and illuminated the skin with different light intensities (Fig. 5b). Strikingly, we observed a concentration and illumination-dependent rescue of both von Frey withdrawal thresholds (Fig. 5c) and dynamic brush or cotton swab-evoked allodynia (Fig. 5d, e) that persisted for more than 2 weeks after a single treatment regime. We examined whether such pronounced effects were also evident in other types of neuropathic pain. Indeed, in both the streptozotocin model of painful diabetic neuropathy^30^, and the paclitaxel model of chemotherapy-induced neuropathic pain^31^, we observed a marked reversal of mechanical hypersensitivity that peaked around 10 days post treatment and returned to injury levels by day 20 (Fig. 5f, g). To determine the selectivity of this approach, we further assessed the effects of BDNF^SNAP^-IR700-mediated photoablation on behavioral responses under basal conditions. We observed no deficits in sensitivity to cold, heat, or pinprick upon treatment (Supplementary Fig. 7A–C). Responses to cotton swab were also unaffected by photoablation (Supplementary Fig. 7D), perhaps because the skin area that is stimulated in this test (50 mm^2^) extends beyond the zone of illumination (15–20 mm^2^).

### Mechanism of BDNF^SNAP^-IR700-mediated ablation

**Fig. 6:**
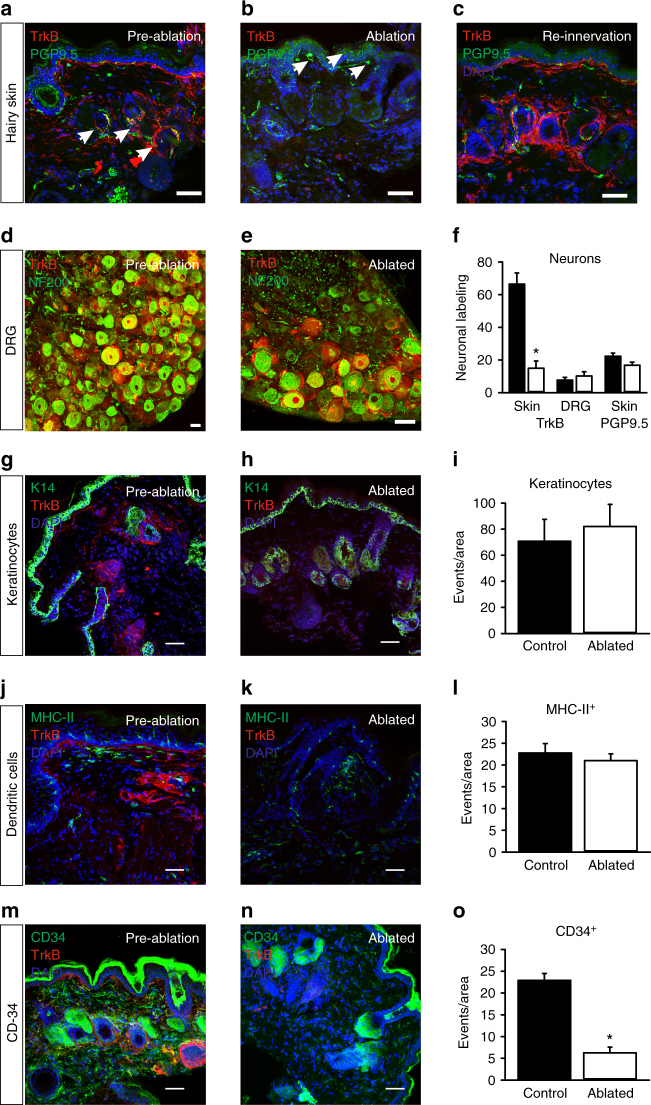
BDNF^SNAP^-IR700 photoablation promotes local retraction of TrkB^+^ afferents. **a**–**c** Substantial loss of TrkB^CreERT2^ positive afferents (red), but persistence of other fibers (green) upon BDNF^SNAP^-IR700-mediated photoablation. **a** Innervation of paw hairy skin prior to ablation, arrows show lanceolate endings. **b** Loss of TrkB^CreERT2^ afferents after ablation, arrows show PGP9.5 fibers. **c** Re-innervation of skin by TrkB^CreERT2^ afferents at 24 days post ablation. **d** DRG section from control TrkB^CreERT2^ mouse labeled for RFP (red) and NF200 (green). **e** DRG section from photoablated TrkB^CreERT2^ mouse labeled for RFP (red) and NF200 (green). **f** Quantification of the proportion of hair follicle innervation and DRG neurons positive for TrkB following photoablation in the paw and the quantification of PGP9.5+ free nerve endings showing the numbers of free nerves remain unaffected. Representative skin sections from control and BDNF^SNAP^-IR700 photoablated mice labeled with the indicated antibodies. TrkB-positive cells are indicated in red and DAPI-positive nuclei in blue. **g**, **h** Keratinocytes labeled with K14 (green). **i** Quantification of the number of K14+ cells. **j**,** k** Dendritic cells and dermal antigen-presenting cells labeled with MHC-II (green) and **l** the quantification for MHC-II^+^ cells. **m**, **n** Mast cells and epithelial and endothelial progenitor cells labeled with CD34 (green). **o** Quantification of the number of K14+ cells. Scale bars, 40 µm. Error bars indicate SEM

Using a TrkB^CreERT2^::Rosa26^SNAPCaaX^ reporter mouse line^28^ to identify TrkB-positive afferents, and a PGP9.5 antibody to label all fibers, we examined the innervation density of hypersensitive skin over the course of phototherapy. Prior to photoablation, we detected TrkB-positive lanceolate endings around hair follicles (Fig. 6a) and innervation of Meissner corpuscles in the plantar surface of the paw (Supplementary Fig. 8D). At 7 days after photoablation (13 days post-SNI) when behavioral reversal of mechanical hypersensitivity was most pronounced, we observed selective loss of TrkB fibers but persistent innervation by PGP9.5 fibers in hairy and glabrous skin (Fig. 6b, f and Supplementary Fig. 8). Indeed, many hair follicles displayed a complete loss of TrkB innervation but still contained PGP9.5-positive circumferential and free nerve endings demonstrating the remarkable specificity of ablation (Supplementary Fig. 9). At 24 days post photoablation when mechanical hypersensitivity had reverted, TrkB-positive fibers were again seen innervating their appropriate end organs in both glabrous and hairy skin (Fig. 6c and Supplementary Fig. 8). Importantly, we observed no apparent reduction in innervation of control tissue injected with unconjugated IR700 and illuminated (Figure Supplementary Fig. 9). We further investigated whether loss of TrkB^CreERT2^ neurons was also evident at the level of the cell soma by analyzing the number of TrkB^CreERT2^-positive neurons in the DRG. We observed no difference in the proportion of TrkB neurons 10 days after photoablation (Fig. 6d–f), indicating that the loss of fibers likely reflects local retraction from their peripheral targets.

TrkB is also expressed by other cells in the skin in addition to sensory fibers^32–35^. We sought to identify these cell types and determine whether they are lost upon photoablation and contribute to the behavioral phenotype. TrkB was not detected in Merkel cells, keratinocytes, or dendritic and dermal antigen-presenting cells (Supplementary Fig. 10 and Fig. 6g, j), and BDNF^SNAP^-IR700-mediated photoablation did not alter their numbers in the skin (Fig. 6h, k, i, l). Expression of TrkB was however evident in cells labeled with CD34, a marker of mast cells and epithelial and endothelial progenitor cells (Fig. 6m). Moreover, photoablation significantly reduced the number of CD34-positive cells in the skin (Fig. 6n–o).

**Fig. 7:**
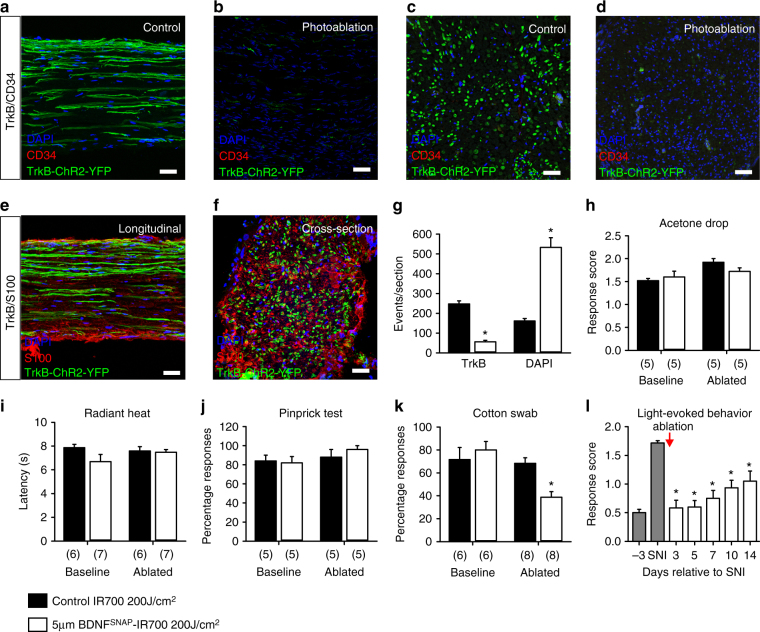
Photoablation of the sciatic nerve shows requirement of TrkB^+^ neurons in mediating allodynia. **a**–**f** Representative images of the sciatic nerve labeled for TrkB in green, DAPI in blue, and CD34 or S100 in red. Longitudinal sections of the nerve show reduction in TrkB^+^ fibers, but no detectable CD34 in **a** non-ablated control and **b** photoablated mice. Cross-section of the sciatic nerve from **c** control and **d** photoablated mice shows reduction in TrkB^+^ fibers. No co-localization between TrkB^+^ fibers and S100+ cells in **e** longitudinal sections and **f** cross-section of the nerve. **g** Quantification of the numbers of TrkB^+^ fibers and DAPI labeled cells in cross sections of sciatic nerve. Behavioral sensitivity following BDNF^SNAP^-IR700-mediated ablation in the sciatic nerve: **h** acetone drop test (*t*-test; *p* = 0.151), **i** radiant heat test (*t*-test; *p* = 0.829), and **j** pin-prick test (*t*-test; *p* = 0.548) are not altered by nerve photoablation. However, sensitivity to **k** cotton swab (*t*-test; *p* = 0.001) in control animals, and **l** light-evoked behavior in TrkB^CreERT2^::Rosa26^ChR2^ mice with SNI, are reduced by nerve photoablation (two-way RM ANOVA; *p* = 0.002). White bars 5 µM BDNF^SNAP^-IR700 at 200 J/cm^2^, black bars 5 µM IR700 at 200 J/cm^2^. Baseline indicates pre-ablation and pre-treatment. Error bars indicate SEM. Scale bars, **a**–**d** 40 μm, **e** 10 μm

To determine whether it is loss of CD34^+^ cells or TrkB^+^ afferents which influences sensory behavior, we injected BDNF^SNAP^-IR700 into the sciatic nerve at mid-thigh level of SNI mice and illuminated the nerve to ablate TrkB sensory fibers but spare CD34 cells in the skin. We first examined histological indicators at the site of injection. In control samples, TrkB^+^ fibers were clearly visible in both longitudinal and cross sections of nerve, whereas after illumination they were essentially eliminated (Fig. 7a–d, g). Importantly we did not detect any CD34^+^ cells in nerve samples, either before or after illumination (Fig. 7a–d). Similarly, TrkB expression was also not evident in other non-neuronal cell type in the sciatic nerve such as S100-positive Schwann cells (Fig. 7e, f). Finally, we quantified the number of DAPI-positive nuclei in nerve sections as a measure of non-neuronal cell density. There was no overlap between DAPI and TrkB expression (Fig. 7a, c, e–g) indicating that TrkB is restricted to neurons in the nerve; indeed, we observed an increase in DAPI-positive nuclei after illumination, likely reflecting immune cell infiltration upon photoablation. Collectively, these data indicate that BDNF targeted photoablation selectively and effectively eliminates TrkB-positive fibers.

We further explored the behavioral consequence of TrkB fiber ablation in the sciatic nerve. In animals which received BDNF^SNAP^-IR700 and illumination of the nerve, behavioral responses to cooling, heating, and pinprick were normal (Fig. 7h–j). However, sensitivity to cotton swab was significantly reduced (Fig. 7k), paralleling the results using genetic ablation of TrkB^+^ neurons. Finally, we investigated whether optogenetically evoked pain behavior in TrkB^CreERT2^::Rosa26^ChR2^ mice with SNI is also reduced by BDNF^SNAP^-IR700 nerve injection and illumination. Upon photoablation of TrkB^+^ fibers in the sciatic nerve we observed a significant reduction in light-driven nocifensive behavior in TrkB^CreERT2^::Rosa26^ChR2^ mice (Fig. 4l). Thus, TrkB^+^ sensory afferents, and not CD34^+^ cells in the skin, likely underlie behavioral sensitivity to light touch under basal conditions and after nerve lesion.

## Discussion

Mechanical allodynia is a cardinal feature of neuropathic pain that is challenging to treat and exerts a substantial societal burden. Here we identify the first relay station in the neuronal pathway that confers pain from gentle touch under neuropathic pain states. We demonstrate that TrkB-positive sensory neurons detect the lightest touch under basal conditions, but after nerve injury are both necessary and sufficient to drive mechanical allodynia. We further describe new technology based upon ligand-mediated delivery of a phototoxic agent to target these neurons and reverse mechanical hypersensitivity in neuropathic pain states.

Our work on the identity of TrkB-positive sensory neurons builds upon previous studies that have reported that TrkB is expressed in two populations of myelinated mechanoreceptors that are differentiated by co-expression of Ret, and form longitudinal lanceolate endings around hair follicles^18,19,36,37^. We demonstrate that TrkB^+^ afferents also innervate Meissner’s corpuscles in the glabrous skin, and that TrkB marks essentially all Aδ-LTMR’s (also known as D-hairs) and RA Aβ−LTMR’s, but no other cutaneous sensory neuron subtype, establishing the TrkB^CreERT2^ mouse line as a powerful tool for investigating the function of these neurons in vivo. Moreover, our histological analysis of human tissue indicates that DRG neurons and skin biopsies of human subjects have comparable molecular profiles to mice, suggesting that TrkB^+^ neurons in humans may also form LTMRs.

To explore the role of Aδ-LTMR’s and RA Aβ−LTMR’s in sensory evoked behavior, we took both a loss-of-function approach using genetic ablation, and a gain-of-function approach using optogenetic activation of TrkB^+^ neurons. We found that TrkB^+^ neurons were required for a behavioral response to the gentlest of dynamic touches, but that their ablation had no influence on responses evoked by thermal or stronger static mechanical stimuli. Similarly, blue-light stimulation of the nape of the neck or the border of the ear in TrkB^CreERT2^::Rosa26^ChR2^ mice elicited a flicking of the ears or the head (Supplementary Movie 2) that was different from previously described wiping or itching in response to pain^38^. It was shown as early as the 1940s that the slightest movement provoked by a cotton bristle is enough to activate D-hairs, and that this may represent sensations of tickle or gentle blowing of air^39^. It is intriguing to speculate that optogenetic activation of TrkB^+^ neurons in the ear may elicit a similar sensation.

Previously, TrkB^+^ D-hairs have been shown to specifically innervate hair follicles in a direction-dependent manner and respond to movement of hair in the caudal-to-rostral direction, suggesting that they may be important for tactile determination of direction^18,40^. We did not determine whether mice had deficits in orientating toward a moving mechanical stimulus in the absence of TrkB^+^ neurons. However, the ablation approach described here would be valuable for investigating the contribution of these neurons toward orientation. In the same light, development of more intricate tests to measure gentle tactile stimulation of the skin will help better understand the behavioral consequences of stimuli transduced by TrkB^+^ neurons.

We further reasoned that manipulation of TrkB^+^ neurons through loss- and gain-of-function experiments would also allow us to identify the peripheral neuron type which inputs mechanical hypersensitivity into the spinal cord. Both nociceptors and LTMRs have been implicated in mechanical allodynia but consensus on the sufficiency and necessity of a specific subpopulation of neurons that conveys this sensation is lacking^10–12,14–16,41^. We found that TrkB^+^ LTMRs were required for hypersensitivity to both punctate and dynamic mechanical stimuli after nerve injury, and that optogenetic activation of TrkB^+^ neurons was sufficient to evoke strong nocifensive behavior. Importantly, we also determined that in the CFA model of inflammatory pain, TrkB^+^ neurons were dispensable for mechanical hypersensitivity. Thus, mechanical pain arising from neuropathy or tissue inflammation is likely mechanistically different and should therefore be treated as a distinct clinical entity. Of note, the neurons marked in the TrkB^CreERT2^ line do not express C-fiber markers such as IB4, CGRP, and Vglut3, but overlap with markers of A-fibers, all of which have previously been implicated in mechanical allodynia^10–12, 14–16, 41^. A future challenge will be to determine whether mechanical hypersensitivity is conveyed by only Aδ-LTMR’s or RA Aβ−LTMR’s fibers, or whether both afferent types are required.

We did not explore in detail the mechanistic basis of how TrkB^+^ neurons provoke mechanical allodynia after nerve injury, focusing instead on developing a potentially translatable tool for its treatment. However, our data do give some insights into events in the spinal cord post-injury. For example, using a TrkB^CreERT2^::Avil^mCherry^ mouse line to label TrkB^+^ afferent projections into the dorsal horn, we detected no gross differences in their termination pattern after nerve injury, arguing against sprouting of LTMR’s from lamina III/IV into lamina II^26^. We did however observe c-fos labeling in lamina I evoked by optogenetic activation of TrkB^+^ afferents in the hindpaw of injured mice, indicating that LTMRs do indeed gain access to pain transmitting neurons in lamina I following nerve injury. Recent evidence suggests that this may arise both through altered gene expression in TrkB neurons^16^ and through a defect in the feed-forward inhibition of interneurons within laminae III/IV^3,5,6,42^. The TrkB^CreERT2^ line and tools described here will allow for further investigations into these mechanisms.

Current clinical options for reducing neuropathic pain include opioids (like morphine and oxycodone), anti-epileptics such as gabapentin, and tricyclic antidepressants^43–45^. These drugs have limited effectiveness, serious safety issues, and long-term use can lead to addiction^46^. Cutaneous receptors in the skin represent an attractive target for novel analgesics, however many potential therapies under development, such as anti-NGF antibodies, and sodium channel blockers are geared toward blocking the function of nociceptors. Our data indicate that LTMRs would be a more appropriate target for alleviating mechanical allodynia, and indeed small-molecule inhibitors that silence mechanoreceptors^47^, or inhibit electrical activity in Aβ-LTMRs^15^ have been shown to be effective at reducing mechanical allodynia in mouse models. Our approach takes this further by utilizing the TrkB ligand BDNF to deliver a photosensitizer directly to TrkB^+^ neurons, selectively targeting those neurons which initiate mechanical allodynia. We show that this leads to long-term reversal of mechanical hypersensitivity across models of traumatic, diabetic, and chemotherapy-induced neuropathy, with minimal effects on other sensory modalities.

Application of BDNF^SNAP^-IR700 to the skin and subsequent illumination led to the local retraction of TrkB^+^ neurons from their end organs, followed by a re-innervation of appropriate targets 3 weeks later that paralleled the return of mechanical allodynia. This was a remarkably selective process, as illustrated by the continued presence of circumferential endings around hair follicles (likely TrkC/Ret-positive field receptors^48^), and free nerve endings in the epidermis. Indeed, it is feasible that ligand-targeted photoablation could also be applied to other subtypes of sensory neurons using different ligands to inhibit other sensations. Beyond the therapeutic potential of such an approach, this may also have value as an experimental tool for exploring the consequences of subtype-specific nerve ablation and the events that lead to regeneration.

BDNF^SNAP^-IR700-mediated ablation also allowed us to address a critical question pertaining to the role of TrkB-expressing cells in the skin and their contribution to allodynia. For example, it has been demonstrated that optogenetic activation of keratinocytes can trigger action potentials in some populations of cutaneous sensory neurons (but not Aδ-LTMRs or RA Aβ−LTMRs) and initiate nociceptive behavior^49^. While we detected no expression of TrkB in keratinocytes, Merkel cells or dendritic cells in the skin, TrkB was observed in mast cells and epithelial and endothelial progenitor cells marked by CD34. Moreover, photoablation reduced the number of CD34^+^ cells in the skin in addition to TrkB^+^ fibers. To determine which of these cell types underlies the behavioral phenotype, we performed photoablation on the sciatic nerve. Importantly, CD34^+^ cells were not evident in the nerve, and photoablation of the nerve produced a phenotype similar to our skin injections, suggesting that CD34^+^ skin cells are not responsible. TrkB has also been reported to be expressed by Schwann cells in the nerve^50^; however, we were unable to detect overlap of TrkB with the Schwann cell marker S100. Further experiments using a transectional approach to limit ChR2 expression to TrkB fibers, or a triple transgenic TrkB^CreERT2^::Avil^iDTR^::Rosa26^ChR2-YFP^ mouse line with which to perform optogenetic activation in the absence of TrkB^+^ fibers, would further clarify the role of afferent fibers versus other cell types in mediating mechanical allodynia. Moreover, investigation of the role of non-neuronal TrkB^+^ cells in mediating allodynia would be required for therapeutic development of BDNF^SNAP^-IR700-mediated ablation.

In summary, here we identify the peripheral neuronal substrate that confers pain from gentle touch under neuropathic pain states. We demonstrate that TrkB marks a population of sensory neurons that normally detect the lightest touch but drive mechanical hypersensitivity after nerve injury. We further describe new technology based upon a phototoxic derivative of BDNF to target these neurons and reverse allodynia in multiple types of neuropathic pain. This approach is analogous to clinically approved capsaicin patches, in which a high concentration of capsaicin is applied to the skin and leads to retraction of nociceptive fibers^51^. Instead, here we target directly the neurons responsible for mechanical allodynia, allowing for local, on-demand treatment of pain through application of light. Further use of this technology and of the genetic tools developed here to manipulate TrkB neurons will now allow precise characterization of the central circuits that gate mechanical pain and transform a normally innocuous sensation into a noxious one.

## Methods

### Animals

A bacterial artificial chromosome (BAC) containing the TrkB mouse locus was obtained from Source Bioscience (RP23-391J8). Individual BAC clones were screened and electroporated with a plasmid conferring competence for lambda RedET-mediated recombineering. A cassette containing a modified CreERT2-pA-Frt-Ampicillin-Frt was inserted in the ATG of trkB, deleting the entire coding region of exon 2 by homologous recombination using the primers: homology to CreERT2: gacgcctggctcagcgtagggacacgcactccgactgactggcactggcagctcgggatgtccaatttactga and homology to FAF: cccaaacatacacctgcctgattcctgaggtggggacaggagaaaaagtaaaaggaactcacgccc tgatagacggtttttcgccctttgacgttgg. Clonal identity was confirmed by PCR and full-length sequencing of the inserted cassette and the ampicillin cassette was removed using bacterial Flp. Purified BAC DNA was dissolved into endotoxin-free TE and injected into pronuclei derived from the hybrid B6D2F1/N strain (Charles River Laboratories) or prepared for intracytoplasmic sperm injection (ICSI). Both methods were successful and produced offspring. To determine the genotype of the mouse, PCR was performed using the following primers: gcactgatttcgaccaggtt (fwd) and gagtcatccttagcgccgta (rev), yielding products of 408 bp.

An Avil^hM3Dq-mCherry^ line was obtained by a knock-in of a Lox-STOP-Lox-hM3Dq-mCherry cassette into the Advilin locus, to replace exon 2 and 66 bp upstream. The targeting construct was transfected into A9 ESCs. Individual ESC clones were screened to identify homologous recombinantion. Southern blot was performed as described previously^22^. DNA was digested with PstI and HindIII and hybridized with 5′ or 3′ probe, respectively. We obtained 9600 bp (wild type) and 6300 bp (targeted) DNA fragments by using the 5′ probe and 7100 bp (wild type) and 6100 bp (targeted) DNA fragments by using the 3′ probe. Positive clones were injected into 8-cell-stage embryos to generate heterozygous mice for the targeted allele. To determine the genotype, PCR were performed using the following primer pairs: gccccgtaatgcagaagaag (fwd), gtgtagtcctcgttgtggga (rev).

For diphtheria toxin-mediated ablation, Avil^iDTR^ mice as described previously^22^ were crossed to TrkB^CreERT2^ to generate TrkB^CreERT2^::Avil^iDTR^ heterozygous mice. Littermates lacking the Cre (referred to as Avil^iDTR^) were used as controls. For optogenetic activation using channelrhodopsin, TrkB^CreERT2^ were crossed to Rosa26^ChR2-YFP^ mice to generate TrkB^CreERT2^::Rosa26^ChR2-YFP^ mice.

For efficient Cre recombination, adult TrkB^CreERT2^ mice (6–8 weeks of age) were injected i.p. with 75 mg/kg of body weight of Tamoxifen (Sigma-Aldrich, T5648) in sunflower seed oil (Sigma-Aldrich, S5007) for 5 consecutive days. Mice were then used for experiments 1–2 weeks after the final tamoxifen injection. All behavioral experiments were performed on male mice. Tissue for histological analysis was taken from male and female mice.

Mice were housed in the Epigenetics and Neurobiology unit, EMBL Rome according to the Italian legislation (Art. 9, 27. Jan 1992, no 116) and experiments were performed under license from the Italian Ministry of Health, and in compliance with the ARRIVE guidelines.

### Immunofluorescence

DRG and spinal cord were dissected from adult mice and fixed with 4% PFA for 2 h or overnight at 4 °C. Tissues were embedded in 12% bovine gelatin (Sigma-Aldrich, G9391) and sectioned at 50 µm and 100 µm, respectively. Sections were then treated with cold 50% ethanol for 30 min and incubated with blocking solution containing 5% serum (goat or donkey) and 0.3% Triton-X in PBS for 30 min and subsequently with primary antibodies in blocking solution overnight at 4 °C. Secondary antibodies were added in blocking solution for 1–2 h and the slides were mounted with prolong gold (Invitrogen, P36930).

For SNAP-tag labeling of the skin, 10 µM of Snap Cell TMR-Star (New England Bioloabs, S9105S) was injected intradermally into the back skin and into the paw of TrkB^CreERT2^::Rosa26^SNAPCaaX^ mice^28^. The skin was dissected after 6 h and cryoprotected in 30% sucrose overnight at 4 °C. Tissues were then embedded in Tissue-Tek O.C.T compound. Forty micrometer thick sections were cut using a cryostat (Leica, CM3050S) and stained with one or more antibodies listed below. We used K14 antibodies to identify keratinocytes^52^, MHC-II antibodies for dendritic and antigen-presenting cells^53,54^, and CD34 for mast cells^55,56^, endothelial and epithelial progenitor cells. For assessing the effect of photoablation using BDNF^SNAP^, skin from TrkB^CreERT2^::Rosa26^SNAPCaaX^ mice were used and processed as described above.

For human DRG/skin staining, 20 µm sections of snap frozen tissues were cut, dried, post fixed with 4% PFA and immunostained as mentioned above. Primary and secondary antibodies were incubated in a solution containing 2% BSA (wt/vol) and 0.01% Tween-20 in PBS. Informed consent was obtained from all subjects.

The following primary antibodies were used: rabbit anti-RFP (Rockland, 600-401-379), 1:200, mouse anti-NF200 (Sigma-Aldrich, N0142) 1:500, mouse anti-CGRP (Rockland, 200-301-D15) 1:500, isolectin GS-B4-biotin XX conjugate (Invitrogen I21414) 1:100, rabbit anti-TH, (Millipore, AB152), 1:1000, rabbit anti-PGP9.5 antibody (Dako, Z5116) 1:200, rabbit anti-S100 (Dako, Z0311) 1:200, goat anti-human HB-EGF (R&D systems, AF-259-NA) 1:50, rabbit anti-cFos (Santa Cruz, Sc-52) 1:1000, rabbit anti-TrkB (ThermoFisher PA5-34026) 1:200, Rabbit anti-Ret (Abcam ab134100) 1:100, Mouse anti-TrkA (ThermoFisher MA5-15509) 1:100, rabbit anti-K14 (Covance PRB-155P) 1:200, PE-anti mouse I-A/I-E (Biolegend 107607) 1:100, CD34-Biotin conjugate (ThermoFisher 13-0341-82) 1:200. All Alexa-conjugated secondary antibodies were used at 1:1000 concentration and streptavidin-conjugated antibodies were used at 1:600 concentration. All images were taken with a Leica SP5 confocal microscope and analyzed in ImageJ.

### In situ hybridization

DRGs were dissected from TrkB^CreERT2^ mice and post fixed overnight in 4% PFA at 4 °C and cryoprotected in 30% sucrose solution overnight at 4 °C. Tissues were then embedded in tissue Tek O.C.T compound and 10 mm sections were cut using a cryostat. In situ hybridization was performed using a riboprobe generated from a cDNA template^57^. Briefly, sections were digested with proteinase K for 6 min, acetylated, and hybridized with the either antisense or sense probes in 50% formamide, 5× SSC, 5× Denhardt’s solution, 500 µg/ml salmon sperm DNA and 250 µg/ml tRNA overnight at 56 °C. After post hybridization, washes were done with 50% formamide, 2× SSC, then 0.5× SSC at 56 °C, and with 2× SSC at ambient temperature, sections were blocked and incubated overnight with anti-digoxigenin-AP (Roche; at 1:1000). Signal detection was done using NBT/BCIP substrate.

### Ex vivo skin nerve preparation

The skin nerve preparation was performed on 8–16 weeks old mice killed with CO_2_ followed by cervical dislocation. After dissection, the hairy and glabrous skin of the hind limb was placed corium side up with the sural nerve attached in the bath chamber. During the recording, the skin was bathed with 32 °C-warm synthetic interstitial fluid (SIF buffer in mM 108 NaCl, 3.5 KCl, 0.7 mM MgSO_4_, 26 mM NaHCO_3_, 1.7 mM NaH_2_PO_4_, 1.5 mM CaCl_2_, 9.5 mM sodium gluconate, 5.5 mM glucose, and 7.5 mM sucrose at a pH of 7.4). The sural nerve was teased into thin bundles and laid on the recording electrode connected to the neurolog extracellular recording amplifier (Digitimer, modules NL104, NL125/NL126). Single fiber units were located by mechanically stimulating different regions of the skin using brush and punctate stimuli. Data were recorded with the Powerlab 2 4SP system and Labchart 7.1 software (AD Instruments) and then the units were classified according to their conduction velocity, von Frey hair thresholds, and adaptation properties to suprathreshold stimuli. Further characterization with ramp-and-hold mechanical stimulation was achieved with a computer-controlled nanomotor (Kleindieck), which allowed for simultaneous recording of electrophysiological and stimulation data. For light stimulation of ChR2-expressing fibers, blue-light pulses from a 470 nm laser were focused on discrete spots by coupling the light guide (FG910UEC, Thorlabs) to a ×20 microscope objective using a custom-made adapter. The different light-pulse durations and frequencies were generated by the built-in stimulator function of LabChart 7.1 and simultaneously recorded. The light intensities were assessed with a powermeter (PM100D, Thorlabs).

### Diphtheria toxin injection

TrkB^CreERT2^::Avil^iDTR^ mice were injected i.p. with 40 μg/kg of diphtheria toxin (Sigma, D0564) twice with a gap of 72 h between the two injections. Behavioral tests were performed 2 weeks after the final DTX injection to avoid potential effects of inflammation. All mice received diphtheria toxin injections.

### Behavioral testing

All behavior experiments were performed on adult male mice (>8–10 weeks of age). For controls, littermates without the Cre were used. The experimenter was always blind to the genotype of the mice.

For testing the effect of evaporative cooling, an acetone drop test was performed. Mice were habituated for 30 min on an elevated platform with mesh flooring. A single drop of cold acetone was sprayed onto to the dorsal or the plantar side of the hindpaw of mice using a syringe without touching the paw of the mice^58^. The behavioral responses were scored according to the following scheme: 0 = no response, 1 = paw withdrawal or a single flick, 2 = repeated flicking of the paw and 3 = licking of the paw. The test was repeated five times by alternating the paw and mice between trials.

To measure heat nociception, mice were placed on top of a hot plate (Ugo Basile, 35150) that was preset to 52 °C and the latency to response as distinguished by flicking or licking of the hindpaw was noted. In order to avoid injury to the mice, a cutoff of 30 s was set.

The latency to respond to heat was measured by focusing thermal heat onto the plantar side of the paw or the tail of the mice using a modified Hargreaves apparatus. In order to avoid injury, a cutoff of 15 s was set.

The grip strength test was performed to test the ability of mice to grip a metal grid (Bioseb; BIO-GS3). The mice were placed on a metal grid with all four paws touching the grid. The tail of the mice was gently pulled and the force at which the mice first let go of the grid was measured. The test was repeated five times with a 30 s interval between trials.

The plantar side of the hindpaw was gently touched using an insect pin glued onto a 1 g von Frey filament without penetrating the skin and the responses were noted for 10 trials with an interval of 1 min between the trials.

Mice were habituated to a plexiglass chamber for 15 min. A 3 cm × 1 cm piece of adhesive tape (Identi tape) was applied along the back of the mice. Responses were quantified as an attempt to remove the tape by shaking, scratching, or biting of the tape over a 5 min period^59^.

Mice were placed on an elevated platform with a mesh floor and habituated for 30 min. A cotton swab was “puffed-out” to about three times its original size. The hindpaw of mice was then brushed using this cotton swab in the heel-to-toe direction and the frequency of responses were noted^60^. The trials were repeated 10 times alternating between paws with an interval of 1 min between trials.

For the von Frey test, mice were habituated on an elevated platform with a mesh floor for 30 min. The plantar side of the hindpaw was stimulated with calibrated von Frey filaments (North coast medical, NC12775-99). The 50% paw withdrawal thresholds were calculated using the Up-Down method^61^. Post SNI, von Frey testing was confined to the sural nerve innervating region of the paw.

The nape of the neck was shaved and stimulated with a 0.07 g von Frey hair, 10 times with an interval of 1 min between stimulation. Frequency of responses as visualized by flicking of the head was measured.

To measure dynamic allodynia, the plantar hindpaw was stimulated by stroking using a paint brush in the heel-to-toe direction. The responses were scored as described by Duan et al.^3^. For each test under baseline conditions, 0 = no response, 1 = paw withdrawal, 2 = flicking of the paw, and 3 = licking of the paw. After injury responses were quantified: 0 = brief withdrawal, 1 = prolonged paw withdrawal toward the body, 2 = repeated flicking or flinching of the affected leg, and 3 = licking of the paw.

### Mouse pain models

The spared nerve injury (SNI) was performed as described by Pertin et al.^62^. Adult mice were anesthetized using 2.5% isoflurane. The sciatic nerve near the thigh region was exposed; the common peroneal and tibial nerves were ligated and cut, leaving the sural nerve intact. Behavioral testing was performed starting from 3 days after injury. Sham controls underwent exposure of the thigh region without any damage to the nerve.

For the CFA model of pain, 20 μl of CFA (Sigma-Aldrich, F5881) was injected into the hindpaw of mice. Behavioral tests were performed 24 and 48 h after inflammation. Ten percent saline injections were used as sham controls

To mimic chemotherapy-induced neuropathic pain, wild-type C57BL6 mice were injected with 1 mg/kg of paclitaxel (Sigma-Aldrich, T7191) intraperitoneally for 4 alternate days^63^. Mechanical thresholds were assayed 1 week after the final injection.

To induce diabetic neuropathy, wild-type C57BL6 mice were injected systemically with 180 mg/kg of Streptozotocin (STZ, Sigma-Aldrich S0130) in 0.05 M citrate buffer (pH 4.5). This led to the development of diabetes within 3 days of administration^64^. The blood–glucose levels were monitored frequently using ACCU-CHEK glucose sticks (Aviva). Mice with blood–glucose levels greater than 350 mg/dl were considered diabetic. The blood–glucose levels were maintained between 350 and 400 mg/dl by administration of insulin (Caninsulin 40 U/ml MSD animal health). Four weeks after the onset of diabetes, mice developed mechanical hypersensitivity. Behavior experiments to test allodynia were carried out as described earlier.

### Light activation using channelrhodopsin

Optogenetic stimulation of the paw was performed using 473 nm Blue DPSS laser (SLOC CO., Ltd; BL473T8) coupled to a powerlab source (Powerlab 4SP) to generate light pulses. Five–10 ms pulses of blue light of (15 Hz) were applied onto the plantar hindpaw of mice using an 0.93NA optic fiber cable (SLOC CO., Ltd; FC-200-10-PC). Paw withdrawal to light application was considered as a positive response. In all experiments, ipsilateral and contralateral paws of TrkB^CreERT2^::Rosa26^ChR2-YFP^ were tested along with littermate controls (referred to as Rosa26^ChR2-YFP^).

### C-fos labeling

For c-fos labeling of the spinal cord, TrkB^CreERT2^::Rosa26^ChR2^ mice were exposed to optogenetic stimulation of the injured (ipsilateral) and contralateral paw with a 15 Hz laser for 1 min. Mice were then killed and the lumbar spinal cord was removed and fixed with 4% PFA overnight at 4 °C. Tissues were then cryoprotected in 30% sucrose and embedded in Tissue-Tek O.C.T compound. Forty micrometer sections of the spinal cord were cut using a cryostat and stained for antibody against c-fos following the protocol described above.

### Production of recombinant BDNF^SNAP^

cDNAs encoding for the murine BDNF and SNAP proteins including a C-terminal poly-Histidine Tag (His_6_) inserted for purification purposes were cloned into the pMP-PB vector as a fusion protein^65^. Protein expression was carried out using Chinese hamster ovary (CHO) cells (ATCC, mycoplasma negative) as described by Balasubramanian et al.^66^. Secreted BDNF-SNAP was purified from cell medium using a Ni-NTA resin (Qiagen, #30210) and eluted with an excess of imidazole. Eluted fractions were then pooled, concentrated, and stored for further analysis.

### Synthesis of BG-IR700

Aliquot of 3 mg of IRDye700DX N-hydroxysuccinimide ester fluorophore (LI-COR Biosciences GmbH, Bad Homburg, Germany) was dissolved in 150 μl DMSO and treated with 1.5 mg BG-PEG11-NH2 and 5 μl diisopropylethylamine. After 1 h, the product BG-PEG11-IRDye700DX was purified by HPLC using a Waters Sunfire Prep C18 OBD 5 µM; 19 × 150 mm column using 0.1 M triethylammonium acetate (TEAA) (pH 7.0) and 0.1 M TEAA in water/acetonitrile 3:7 (pH 7.0) as mobile phases A and B, respectively. A linear gradient from 100% A to 100% B within 30 min was used. The fractions containing the product were lyophilized^67^.

### In vitro labeling of BDNF-SNAP

HEK293T cells (ATCC, mycoplasma negative) were transfected with a combination of 0.125 µg trkB/trkA/trkC in pcDNA plasmid and 0.125 µg p75NTR (all gifts from Moses Chao, Addgene plasmid #24088, #24089, #24093, and #24091, respectively) using Lipofectamine 2000 (Invitrogen, 11668-019) in a medium containing DMEM (Gibco, 41966-029), 10% FBS, 1% penicillin/streptomycin. Before labeling, cells were incubated in serum-free medium for 1 h. Aliquot of 0.1 µM BDNF^SNAP^ was coupled to 0.3 µM of SNAP-surface 647 (New England Biolabs, S9136S) for 1 h at 37 °C and applied onto cells for 15 min at 4 °C and imaged using a Zeiss AxioObserver A1 microscope.

DRG from TrkB^CreERT2^::RFP mice was collected in PBS and incubated in 1 mg/ml collagenase IV (Sigma-Aldrich, C5138) and 0.05% Trypsin (Gibco, 25300-054) for 25 min each at 37 °C. Cells were filtered and suspended in medium containing DMEM (Gibco, 41966-029), 10% heat inactivated fetal bovine serum (PAA, A15101), 0.8% glucose, and 100 U of penicillin/streptomycin (Gibco, 15140-122). Cells were plated on glass coverslips treated with poly-L-lysine and stored at 37 °C. An equimolar concentration of BDNF^SNAP^ was coupled to a mixture of SNAP-Biotin (New England Biolabs, S9110S) and QD655 quantum dots (Invitrogen, Q10121MP) at 37 °C for 30 min. Cells were labeled with the above mixture for 5 min and imaged using a Zeiss AxioObserver A1 microscope.

### In vitro photoablation

HEK293T cells transfected with TrkB (as described above) were incubated with a mixture of 1 µM BDNF^SNAP^ and 3 µM BG-IR700 for 30 min at 37 °C. Cells were then exposed to near infra-red light (680 nm) illuminated at 40 J/cm^2^ for 2 min. Twenty-four hours after light exposure, cells were stained with propidium iodide (eBioscience; 00-6990-50) to assess cellular apoptosis. Controls used were non-transfected cells with BDNF^SNAP^-IR700 and transfected cells with IR700 alone.

### In vivo photoablation

The left hindpaw of C57BL6 mice was injected with different concentrations of BDNF^SNAP^ coupled to BG-IR700 (1:3). Fifteen–twenty minutes after the injections, near infra-red light (680 nm) at 120–150 J/cm^2^ or at 550–600 J/cm^2^ was applied onto the paw of the mice for 4–15 min. This procedure was repeated for 3 consecutive days. Behavioral tests were performed 1 day after the final treatment. For rescue experiments, photoablation was performed following robust development of neuropathic pain as mentioned above. For the in vivo photoablation of the sciatic nerve, the nerve was first exposed at the mid-thigh level; 5 µM BDNF^SNAP^ coupled to BG-IR700 (1:3) was injected directly into the nerve. Ten minutes after the injection, near infra-red light (680 nm) at 200 J/cm^2^ was applied onto the nerve for 45 s.

### Quantification of cell types in skin

To quantify PGP9.5-positive free nerves in the skin, individual intra-epidermal fibers were identified and the number of fibers per section of skin was counted. Similarly, total number of K14 and MHC-II-positive cells was identified using DAPI labeling of nuclei. For CD34, number of TrkB/CD34-positive cells was quantified over an area of 1 mm^2^.

### Statistical analysis

All statistical data are presented as standard error of the mean (SEM) along with the number of samples analyzed (*n*). Student’s *t-*test and/or two-way repeated measures ANOVA were used and *p* < 0.05 was considered statistically significant. Sample sizes were determined from the power of the statistical test performed. No animals were excluded and all experiments were performed blinded with order of testing randomized.

### Data availability

The data that support the findings of this study are available from the authors on reasonable request.

## Electronic supplementary material

Supplementary Information

Description of Additional Supplementary Files

Supplementary Movie 1

Supplementary Movie 2

Supplementary Movie 3
