# Supplementary material for: Control of mechanical pain hypersensitivity in mice through ligand-targeted photoablation of TrkB-positive sensory neurons
Source: Nat Commun. 2018 Apr 24;9:1640. doi: 10.1038/s41467-018-04049-3 (PMC5915601; doi:10.1038/s41467-018-04049-3)
Supplement: Supplementary file 1 — Supplementary Information [file 41467_2018_4049_MOESM1_ESM.pdf]

Supplementary Information

Control of mechanical pain hypersensitivity in mice through ligand-targeted photoablation of TrkB positive sensory neurons

Dhandapani et al.

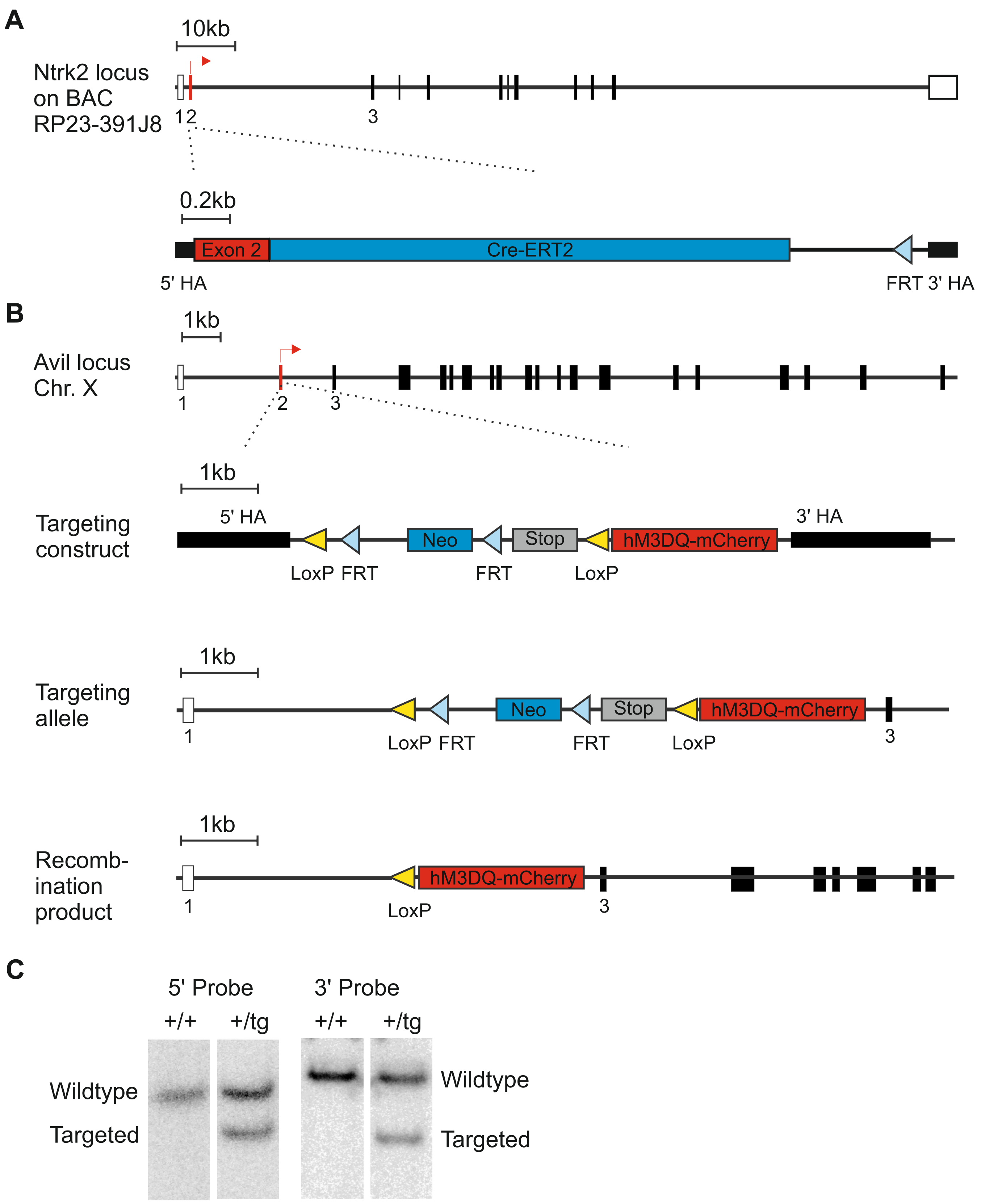

Supplementary Figure 1. Generation of TrkB^{CreERT2} and Avil^{mCherry} mouse lines.

(A) Schematic representation of ET-recombination based insertion of a Cre^{ERT2} gene cassette into the coding region of a BAC containing the mouse TrkB gene locus **(B)** Schematic diagram of the wild type *Avil* locus with the Avil$^{hM3Dq\text{-}mCherry}$ targeting construct, targeted allele and recombination product. **(C)** Southern blot of positive ES clone

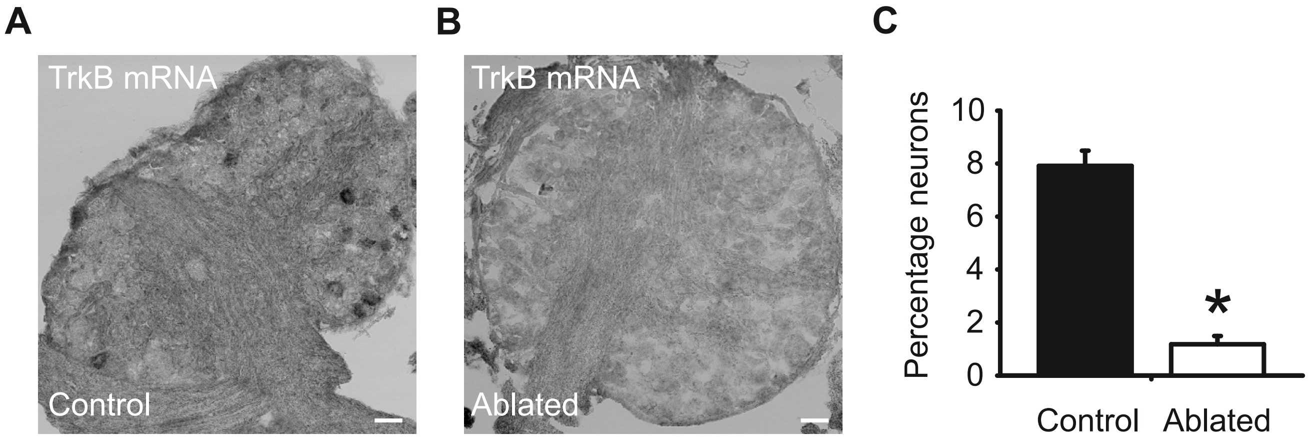

Supplementary Figure 2. TrkB mRNA expression in DRG from control and ablated mice. **(A)** In situ hybridization showing expression of TrkB mRNA in ~8% of neurons in DRG sections from control mice. **(B)** In situ hybridization showing substantial reduction in TrkB mRNA positive cells following diphtheria toxin mediated ablation. **(C)** Quantification of TrkB mRNA positive neurons in control and ablated mice (t-test; $p<0.05$). Scale bars, 50μm. Error bars SEM.

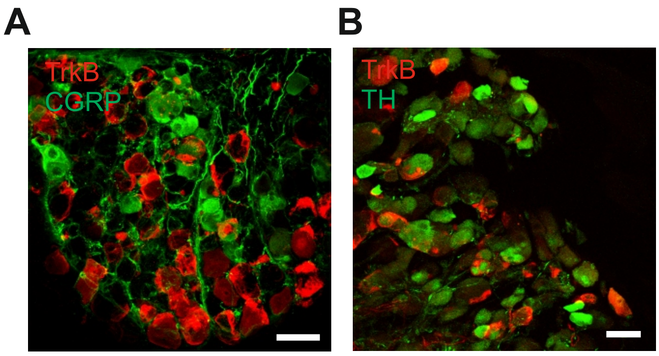

Supplementary Figure 3. TrkB positive sensory neurons are not expressed in nociceptors.

Double immunofluorescence of DRG sections from TrkB^{CreERT2}::Rosa26^{RFP} mice showing that TrkB does not co-localize with **(A)** CGRP, or **(B)** TH.

A

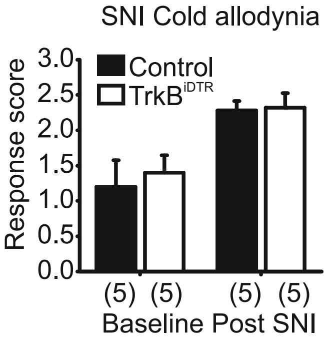

Supplementary Figure 4. Cold allodynia unaffected upon ablation of TrkB+ neurons

(A) Cold hypersensitivity as assayed using the acetone drop test does not differ between $TrkB^{CreERT2}$::$Avil^{iDTR}$ and $Avil^{iDTR}$ control mice (t-test; $p>0.05$).

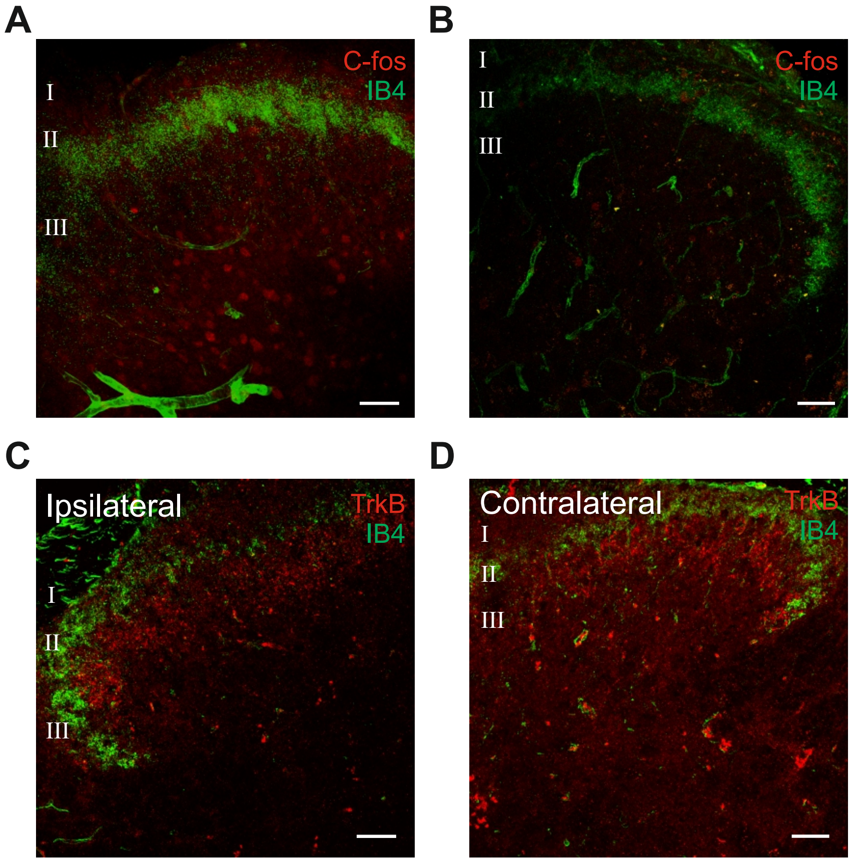

Supplementary Figure 5. C-fos and primary afferent TrkB expression in spinal cord sections.

(A) C-fos expression in spinal cord sections from sham operated $TrkB^{CreERT2}$::$Rosa26^{ChR2}$ mice with light stimulation. **(B)** C-fos expression in spinal cord sections from control $Rosa26^{ChR2}$ mice stimulated with light at 7 days post SNI. **(C and D)** $TrkB^{+}$ primary afferent distribution in $TrkB^{CreERT2}$::$Avil^{mCherry}$ mice at 7 days post SNI. Spinal cord ipsilateral to the injury is shown in **(C)** and contralateral in **(D)**. Scale bars, 40μm.

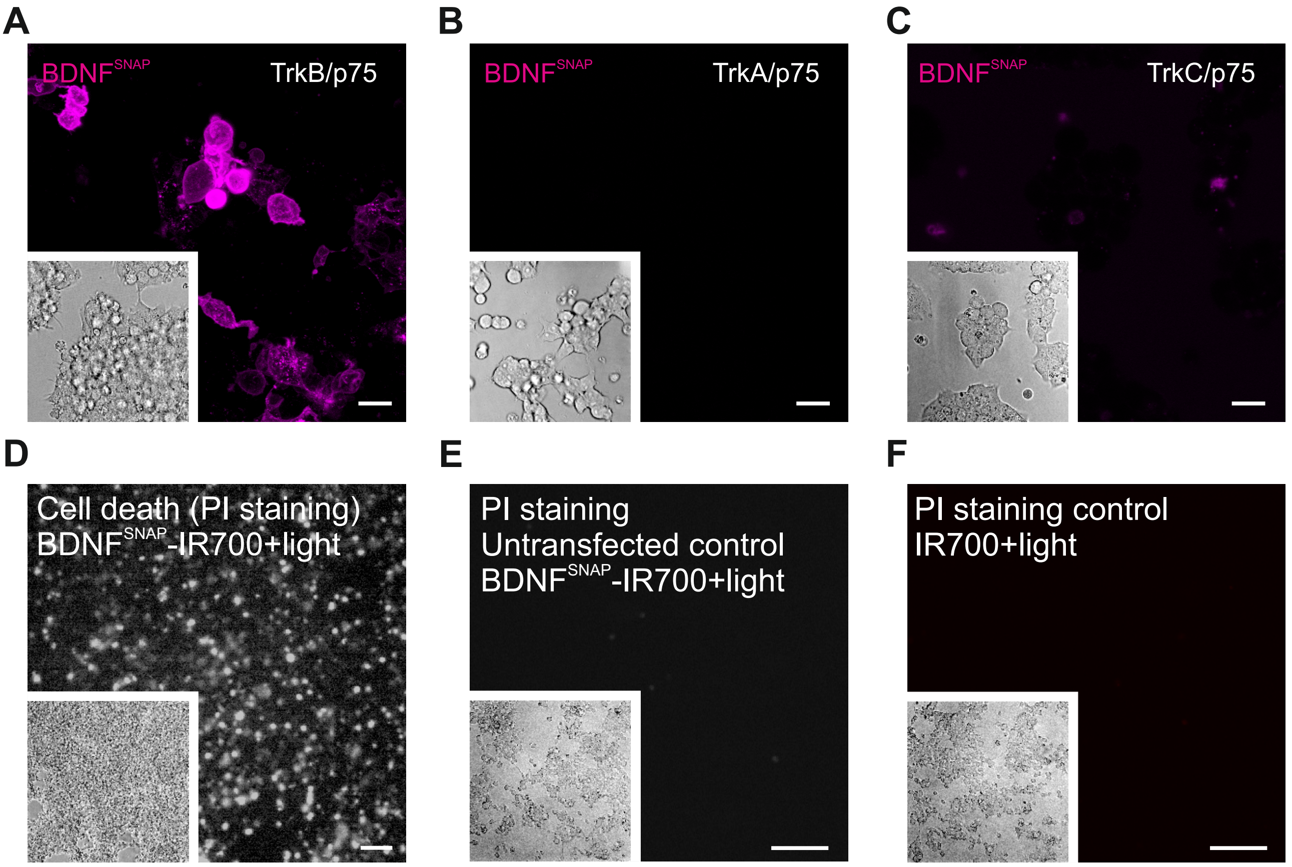

Supplementary Figure 6. BDNF^{SNAP} labelling and IR700 mediated photoablation in vitro.

(A-C) BDNF^{SNAP} labeling of HEK293T cells transfected with **(A)** TrkB/p75NTR, **(B)** TrkA/p75NTR, or **(C)** TrkC/p75NTR. **(D)** Staining of HEK293T cells transfected with TrkB/p75NTR with propidium iodide 24 hours after treatment with BDNF^{SNAP}-IR700 and near infrared illumination. **(E)** Staining of mock transfected HEK293T cells with propidium iodide 24 hours after photoablation following treatment with BDNF^{SNAP}-IR700. **(F)** Staining of HEK293T cells transfected with TrkB/p75NTR with propidium iodide 24 hours after treatment with IR700 alone and near infrared illumination. Scale bars 50µm.

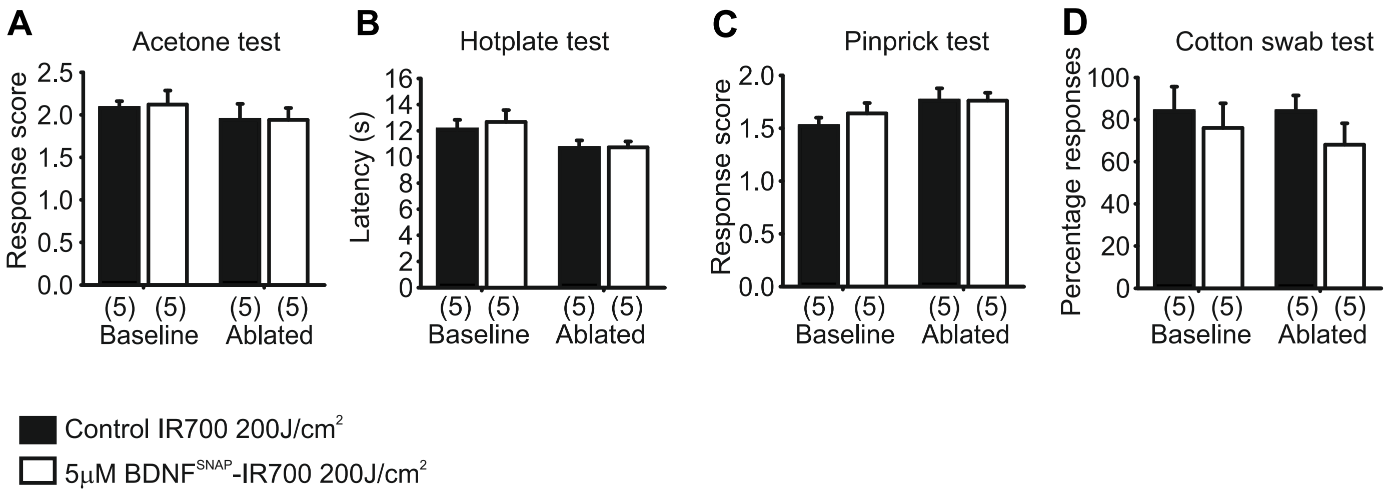

Supplementary Figure 7. BDNF^{SNAP}-IR700 mediated photoablation in the paw does not affect baseline sensory behavior

Responses to **(A)** acetone drop test (t-test; $p>0.05$), **(B)** hot plate test (t-test; $p>0.05$), **(C)** pin-prick test (t-test; $p>0.05$) and **(D)** cotton swab test (t-test; $p>0.05$). White bars 5μM BDNF^{SNAP}-IR700 at 200J/cm^2, black bars 5μM IR700 at 200J/cm^2. Baseline indicates pre-ablation and pre-treatment. Error bars indicate SEM.

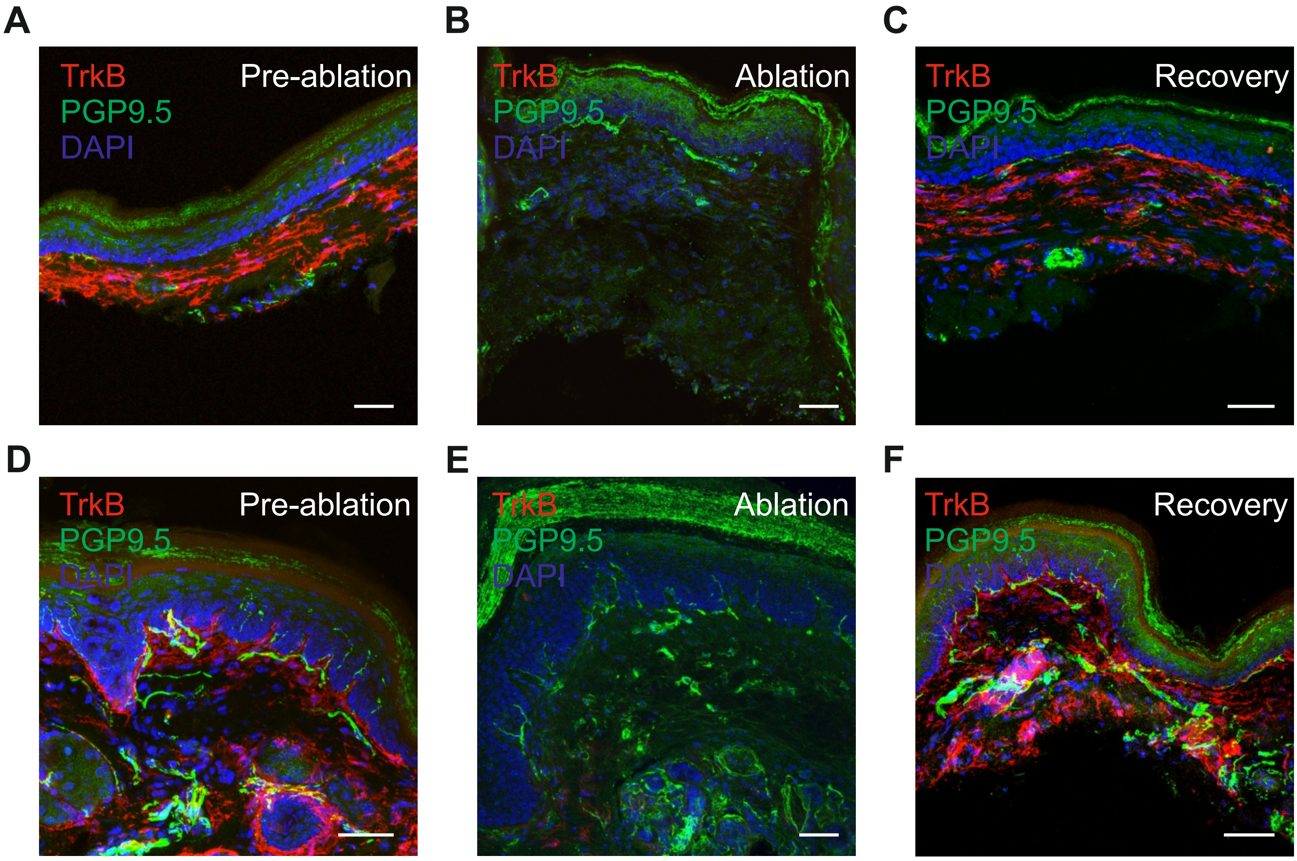

Supplementary Figure 8. Innervation of the plantar skin of the paw upon BDNF^{SNAP}-IR700 mediated photoablation.

(A) Glabrous skin section in the center of the paw prior to photoablation. Note the large number of TrkB^{CreERT2} and PGP9.5 positive fibers that transition through this area. **(B)** Loss of TrkB^{CreERT2} fibers upon ablation. PGP9.5 fibers are still present. **(C)** Recovery of TrkB^{CreERT2} innervation 24 days post ablation. **(D)** Glabrous skin section at the lateral edge of the paw with Meissner's corpuscles prior to photoablation are from 3. Note the dense innervation of Meissner corpuscles by TrkB^{CreERT2} fibres. **(E)** Loss of TrkB^{CreERT2} fibers but not PGP9.5 free nerve endings upon ablation. **(F)** Recovery of TrkB^{CreERT2} fibers 24 days after ablation and reinnervation of Meissner corpuscles. Scale bars 40µm.

A

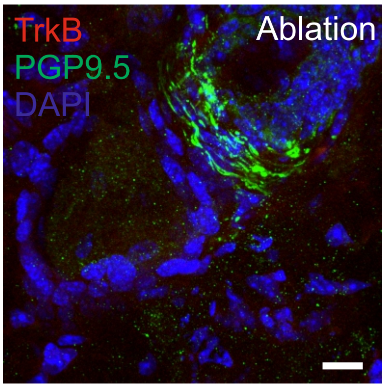

Supplementary Figure 9. Specificity of BDNF^{SNAP} mediated photoablation

(A) High magnification image of a hair follicle after ablation. Note the absence of TrkB^{CreERT2} fibers (red) but PGP9.5 positive circumferential and longitudinal lanceolate endings (green).

A

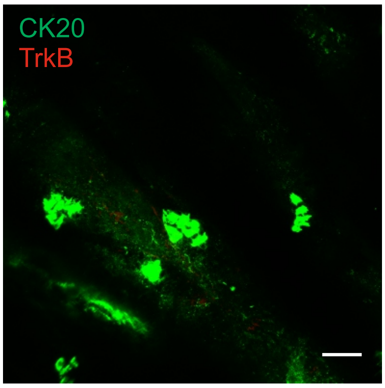

Supplementary Figure 10. Merkel cells from adult mice are TrkB-Cre negative.

(A) Representative image showing Merkel cells (green) labelled with an antibody again CK20. TrkB staining (red) is not present. Scale bar 40µm.

Supplementary Table 1. Quantification of TrkB+ neuronal ablation.

Control non ablated				After ablation			
TrkB	NF200	TrkB+NF200	Total	TrkB	NF200	TrkB+NF200	Total
13	28	5	240	0	15	0	185
5	10	1	79	4	10	0	155
5	13	5	112	3	33	0	85
18	35	11	105	4	26	0	103
7	22	4	62	2	29	0	184
9	23	6	68	0	22	0	146
8	20	3	80				
13	36	7	137				
18	20	3	75				
10	20	2	50				
13	20	5	70				
10	30	4	98				
18	50	12	165				
10	25	5	80				
**157**	**352**	**73**	**1421**	**13**	**135**	**0**	**858**

Numbers of TrkB and NF200 positive DRG neurons in control and diphtheria toxin treated mice.
