# Supplementary material for: Control of mechanical pain hypersensitivity in mice through ligand-targeted photoablation of TrkB-positive sensory neurons
Source: Nat Commun. 2018 Apr 24;9:1640. doi: 10.1038/s41467-018-04049-3 (PMC5915601; doi:10.1038/s41467-018-04049-3)
Supplement: Supplementary file 2 — Description of Additional Supplementary Files [file 41467_2018_4049_MOESM2_ESM.pdf]

Description of Additional Supplementary Files

File Name: Supplementary Movie 1
Description: **Quantification of TrkB+ neuronal ablation.** Numbers of TrkB and NF200 positive DRG neurons in control and diphtheria toxin treated mice.

File Name: Supplementary Movie 2
Description: **Response to optogenetic activation of the hindpaw after CFA.** Video showing the response of a $TrkB^{CreERT2}$::$Rosa26^{ChR2}$ mouse before and after injection of CFA.

File Name: Supplementary Movie 3
Description: **Response to optogenetic activation of the hindpaw after SNI.** Light activation evokes strong paw withdrawal 7 days after SNI in $TrkB^{CreERT2}$::$Rosa26^{ChR2}$ mice, while under baseline conditions, mice do not respond to light.
